# Carotid Endothelial VCAM-1 Is an Early Marker of Carotid Atherosclerosis and Predicts Coronary Artery Disease in Swine

**DOI:** 10.4236/jbise.2015.811075

**Published:** 2015

**Authors:** I. Masseau, D. K. Bowles

**Affiliations:** 1 Department of Biomedical Sciences, University of Missouri, Columbia, MO, USA; 2 Département des Sciences Cliniques, Faculté de Médecine Vétérinaire, Université de Montréal, Québec, Canada; 3 Dalton Cardiovascular Research Center, University of Missouri, Columbia, MO, USA

**Keywords:** VCAM-1, Endothelial Cells, Carotid Artery, Atherosclerosis

## Abstract

**Objective:**

The aim was to determine if endothelial VCAM-1 (eVCAM-1) expression in the common carotid artery (CCA) would correlate with predictive markers of atherosclerotic disease, would precede reduction of markers of endothelial cell function and would predict coronary artery disease (CAD).

**Methods and results:**

Carotid arterial segments (bifurcation, proximal and distal CCA) were harvested from 14 and 24 month-old male castrated familial hypercholesterolemic (FH) swine, a model of spontaneous atherosclerosis. Quantification of local expression of eVCAM-1, intimal macrophage accumulation, oxidative stress, intima-media (I/M) ratio, intima-media thickness (IMT), endothelial nitric oxide synthase (eNOS) and phosphorylated eNOS (p-eNOS) in selected regions of the carotids revealed a relationship between local inflammation and atheroscle-rotic plaque progression. Importantly, inflammation was not uniform throughout the CCA. Endo-thelial VCAM-1 expression was the greatest at the bifurcation and increased with age. Finally, eV-CAM-1 best estimated the severity of CAD compared to blood levels of glucose, hypercholestero-lemia, carotid IMT, and p-eNOS.

**Conclusion:**

Our data suggested that eVCAM-1 was closely associated with atherosclerotic plaque progression and preceded impairment of EDD. Thus, this study supported the use of carotid VCAM-1 targeting agents to estimate the severity of CAD.

## 1. Introduction

Inflammation is increasing recognized as a major contributor to the initiation and progression of atherosclerosis. Hypercholesterolemia leads to endothelial expression of adhesion molecules such as vascular cell adhesion molecule-1 (VCAM-1) at atheroprone areas such as curvatures and bifurcations [1]. Transmigration, accumulation and differentiation of inflammatory cells into foam cells increase intima-media thickness (IMT), a clinical marker of atherosclerosis [2]. Foam cell derived reactive oxygen species in uncouple endothelial nitric oxide synthase (eNOS), decreases nitric oxide bioavailability, and impairs endothelial-dependent dilation (EDD).

In humans, impaired brachial artery flow-mediated dilation (FMD), a non-invasive measure of EDD, can predict future cardiovascular events [3] [4] and the presence of distant (*i.e.* coronary artery) disease despite the absence of local (*i.e.* brachial artery) disease [5] [6]. Furthermore, abnormal coronary vasomotor responses to acetylcholine infusion may be obtained in the absence of measurable disease [7] by providing evidence that impaired EDD precedes disease. However, limitations to utilize EDD as a diagnostic predictor of CAD include poor correlation between FMD and severity of CAD [5] and the inability to detect CAD in patients with dysli-pidemia [8]. In addition, atherosclerotic plaque has been shown to develop in peripheral arteries despite normal EDD [9]. Thus, overall the evidence remains equivocal whether impaired EDD precedes or follows the appearance of disease.

The purpose of this study is to determine if inflammation, *i.e.* VCAM-1 expression, is a better diagnostic tool of early plaque development than other markers of atherosclerosis, including EDD, and in addition if peripheral VCAM-1 can predict the extent of CAD. We first evaluate the direct association between local VCAM-1 expression and markers of severity of disease and endothelial cell function in selected regions of the carotid arteries. We hypothesize that VCAM-1 expressed along the carotid endothelium will be correlated with intimal macrophage infiltration, oxidative stress, IMT, and intima-media ratio (I/M). Additionally, expression of VCAM-1 will precede the reduction of markers of endothelial cell function such as phosphorylated- and total-eNOS in the carotid arteries.

Second, we determine if inflammation, as reflected by endothelial VCAM-1 expression, is uniformly distributed along the common carotid artery (CCA). As for local inflammation, we evaluate the association between endothelial VCAM-1 in the bifurcation, proximal and distal CCA, and markers of severity of disease and of en-dothelial cell function. We hypothesize that endothelial VCAM-1 will be elevated in the bifurcation compared to the other carotid segments, along with markers of disease severity and inversely correlated with markers of en-dothelial cell function.

Third, we compare carotid endothelial VCAM-1 and risk factors for atherosclerosis, such as hyperglycemia, hypercholesterolemia, hypertriglyceridemia, high low-density lipoprotein cholesterol (LDL-C), and low high- density lipoprotein cholesterol (HDL-C), to determine which of these parameters constitutes the best predictor of CAD. We hypothesize that carotid inflammation will better predict CAD compared to other risk factors and markers of endothelial cell function.

These hypotheses are tested using the Familial Hypercholesterolemic (FH) swine model developed by Rapacz and collaborators [10]. This model of atherosclerosis is chosen because these animals develop spontaneous advanced coronary lesions which are similar to those seen in humans [10]. We use FH pigs of two different ages, 14 and 24 months old, to maximize the range of carotid and coronary artery disease present in these animals.

## 2. Materials and Methods

### 2.1. Ethics Statement

Experimental protocols were approved by the University of Missouri Animal Care and Use Committee and in accordance with the “Principles for the Utilization and Care of Vertebrate Animals used in Testing, Research and Training.”

### 2.2. Experimental Design

Twenty-five male-castrated Rapacz Familial Hypercholesterolemic (FH) swine, a model that develops spontaneous advanced coronary lesions similar to humans [10], were purchased from the University of Wisconsin Swine Research and Teaching Center. Pigs were individually housed in rooms maintained at 20 °C – 23 °C with a 12:12-h light-dark cycle and had ad libitum access to water. Pigs were fed 800 g/day of a high-fat, high-choles- terol diet (relative kcal; 13% protein, 39.6% carbohydrate, 47.4% fat and 2% cholesterol by weight) for 6 months prior to sacrifice. Fifteen 14 month-old (body weight (BW): 49.9 to 82.7 kg) and ten 24 month-old (BW: 44.5 to 171.8 kg) animals were used to maximize the range of atherosclerosis. One week prior to euthanasia, seven 14-month old and five 24-month old swine underwent dual energy X-ray absortiometry (DEXA) scanning under sedation to assess body composition.

#### Immunohistochemistry and immunostaining

At sacrifice, the right coronary (RCA), left anterior descending (LAD) arteries and proximal and distal segments of the left common carotid artery (CCA), including the bifurcation from the brachiocephalic trunk, were collected, cleared from fat and connective tissue and immersed into 10% formalin. Paraffin-embedded sections were stained for eNOS phosphorylated at serine residue 1177 (1:800, BD Transduction Labs), total eNOS (1:800, BD Tranduction Labs), VCAM-1 (no dilution, hybridoma6G10, ATCC), SRA-E5 (1:100, Trans Genic Inc.), and nitrotyrosine (1:400, Chemicon) using a LSAB+ kit from Dako. Photomicrographs of the bifurcation, proximal and distal CCA were captured at 10x magnification using Olym-pus MicroSuite Biological Suite Software connected to an Olympus BX61 motorized system microscope (Leeds Precision Instruments, Minneapolis, MN). Selected regions corresponding to High- and Low-VCAM-1 were visualized with an Olympus BX60 microscope (Leeds Precision Instruments, Minneapolis, MN) and captured using Spot Advanced Software (Version 4.6, Diagnostic Instruments, Sterling Heights, MI).

#### Immunostaining quantification

VCAM-1, Scavenger-Receptor A (SRA), nitrotyrosine, phospho-eNOS and eNOS staining were analyzed using Image-Pro Plus Software (Version 6.2, Media Cybernetics Inc., Bethesda, MD). VCAM-1-stained sections obtained from the bifurcation of 24 month-old swine with the most severe carotid atherosclerotic disease were subjectively evaluated for areas of strongest (High-VCAM-1) and weakest (Low-VCAM-1) endothelial VCAM-1 positive staining. Each area (High- and Low-VCAM-1) was then captured at high magnification (40X). VCAM-1 staining along the endothelium and within the subendothelial area was then quantified as the percent of positive staining per area and sum of the intensity of the positive staining per area. Next, regions corresponding to High- and Low-VCAM-1 were identified on consecutive sections stained for nitrotyrosine, SRA, phospho-eNOS and eNOS and images of these regions (two per arterial section) were captured at high magnification (40×). Percent positive staining and intensity for SRA and nitrotyrosine was determined as the sum of positively stained areas included within the entire image divided by the total area and the sum of the intensity of positive staining per area, respectively. Positive staining for phospho-eNOS and eNOS was quantified as the percent of endothelial positive staining per endothelial area and the sum of the intensity of positive staining per endothelial area. To determine if endothelial VCAM-1 expression is uniformly distributed along the common carotid artery (CCA), we quantified positive staining for each marker as the sum of positively stained endothelial areas divided by the luminal circumference of each arterial section (bifurcation, proximal and distal CCA). We used the luminal circumference for normalization of our positive staining since only the endothelial layer was included in our area used for quantification thus keeping the depth constant. This process allowed us to compare endothelial staining throughout the carotid artery despite the great variation in luminal diameter. Endothelial positive staining for nitrotyrosine, phospho-eNOS and eNOS was quantified similarly to VCAM-1. SRA staining was quantified in the bifurcation, proximal and distal CCA by determining the percent of intimal area positively stained.

#### Morphology

IMT, intimal and medial areas were measured on Verhoeff-Van Gieson (VVG) stained sections using standard planimetery. In brief, each arterial section was captured on Olympus BX61 microscope as previously described for other markers. IMT was defined as the distance between the external elastic lamina and the internal luminal border of the artery. IMT was measured three times at the widest part of the arterial section and averaged. Intimal area was defined as the area between the internal elastic lamina (IEL) and the luminal border of the artery while the area between the IEL and external-elastic lamina (EEL) was referred to as the medial area. Intima-media ratio (I/M) was calculated as the ratio of intimal- over medial-areas. Determination of I/M in the coronary arteries was performed using the same landmarks as for the carotid arteries using NIH Image J software (Bethesda, MD).

Intimal-thickness (IT) and IMT in each region corresponding to High- and Low-VCAM-1 were measured from VVG-stained regions of the bifurcation captured with Spot Advanced Software. Intimal-thickness (IT) was defined as the distance between the internal luminal border and the IEL. Each parameter was measured three times and averaged.

#### Dual energy X-ray absortiometry (DEXA) scan

The animal was placed supine on the DEXA table in a fixed position and scanned once. Body composition was determined by an experienced technician using a computer software (QDR Software for Windows XP, Version 12.4, Hologic Inc., Bedford, MA). Percent body fat was expressed as a ratio of fat mass (g) divided by the total mass (g) × 100.

#### Blood analysis

Standard chemistry profiles were obtained from blood collected the day of DEXA scanning, when available (*n* = 12) or prior to sacrifice (*n* = 13). Triglycerides, LDL-C and HDL-C and total cholesterol content was determined from frozen plasma collected the day of euthanasia (*n* = 22). Three animals died shortly after induction and before blood collection. Results from FH swine were compared to those of five non-FH domestic swine (control group).

#### Statistical analysis

Clinical characteristics of the animals studied were compared using two-tailed Student’s t test for parametric data or Mann-Whitney rank sum test for non-parametric data. Staining, IT, IMT, and I/M were compared for age and origin of segments using 2-way ANOVA with post hoc testing of individual comparisons. Prediction of inflammation and CAD were performed using forward stepwise regression. Linear regression was used to examine relation between two factors. R^2^ values are provided as prediction, rather than correlation was the objective. Statistical analyzes were performed with Sigma Stat Version 3.5 for Windows (Dundas Software, Erkrath, Germany) except linear regression for which GraphPad Prism 5.0d for Mac OS X (GraphPad Software, San Diego, California) was used.

## 3. Results

### eVCAM-1 is a local indicator of atherosclerosis

**Figure 1:**
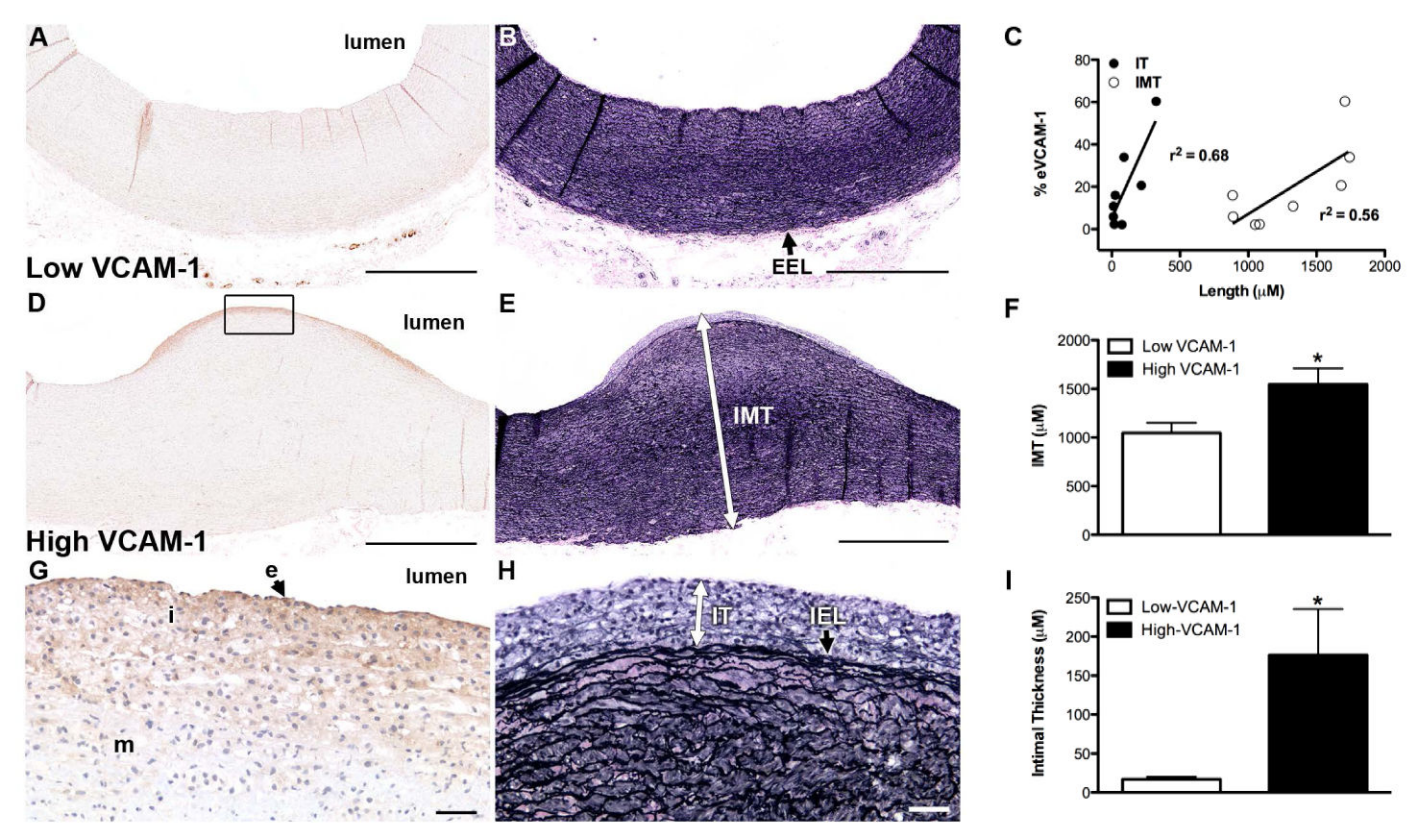
Relationship between local eVCAM-1, intimal thickness and intima-media thickness. Regions with the strongest (High-) and weakest (Low-) VCAM-1 staining were selected in each carotid bifurcation of 24-month-old FH pigs ((A) and (D), respectively). Corresponding Verhoeff-Van Gieson (VVG) stain in regions ((B) and (E)) used for morphology (intima-media thickness (IMT) and intimal thickness (IT)). (G) and (H), enlarged images for VCAM-1 and corresponding VVG staining in the intimal and superficial medial layers from the High-VCAM-1 region in (D). In selected regions of High-VCAM-1, positive staining occupies a greater proportion of the endothelium (E) and is more intense along the endothelium (E). VCAM-1 staining gradually decreases in intensity and distribution through the intima (I) and underlying media (m). (C) Percent endothelial VCAM-1 staining in regions of High- and Low-VCAM-1 was significantly correlated to IT (closed circle) and IMT (open circle). High VCAM-1 regions had a significant increase in IMT (F) and IT (I) compared to regions with low-VCAM-1 staining. Mean values ± SEM are represented (^*^P < 0.05). Scale bar for (A), (B), (D), (E) corresponds to 200 μM; insets ((G), (H)) = 50 μM. eVCAM-1, endothelial VCAM-1; EEL, external elastic lamina; IEL, internal elastic lamina.

**Figure 2:**
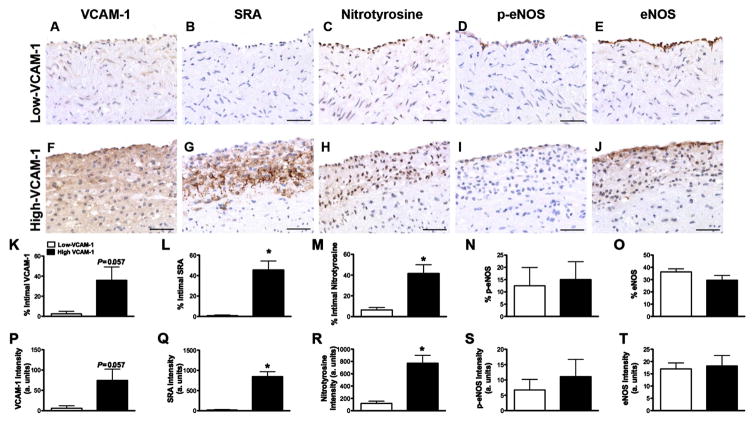
Relationship between eVCAM-1 expression and local progression of atherosclerosis. Representative regions of Low-((A)-(E)) and High-VCAM-1((F)-(J)) stained for VCAM-1, macrophage infiltration (SRA), oxidative stress (nitrotyrosine), phosphorylated eNOS, and eNOS in the carotid bifurcation of a 24-month-old FH pig. (K), (P): As expected, regions selected as High-VCAM-1 had a greater percent area of their intimal area stained for VCAM-1 as well as a more intense positive staining compared to Low-VCAM-1 regions. Regions of Low-VCAM-1 had minimal positive staining for macrophage infiltration ((L), (Q)) or oxidative stress ((M), (R)) compared to high- VCAM-1 regions. In these regions, clusters of positive staining for SRA were seen predominately within the intima while nitrotyrosine staining was seen along the endothelium and diffusely throughout the intima. (D) (I): Positive staining for phosphorylated eNOS at ser-1177 is limited to the endothelium. The endothelium covering both regions of high and Low-VCAM-1 was stained similarly. However, eNOS staining was present in the intimal region as well as along the endothelium in High-VCAM-1 region (J). The percent of endothelial positive staining or intensity for p-eNOS (N, S) or eNOS (O, T) were not different between regions of High- and Low-VCAM-1. Scale bar corresponds to 50 μM. (K)-(T): Mean values ± SEM are represented (^*^P < 0.05). SRA: Scavenger Receptor A.

To determine if VCAM-1 is a good local indicator of carotid atherosclerosis, we evaluated the bifurcation of four 24-month old pigs with the greatest amount of disease. Within each cross-section, we selected one region with minimal (Low-VCAM-1) and one region with maximal (High-VCAM-1) eVCAM-1 staining (Figure 1(A) and Figure 1(D), respectively). We then measured IMT and IT as illustrated (Figure 1(E) and Figure 1(H)) using corresponding VVG regions. VCAM-1 was significantly correlated with IT (*r*^2^ = 0.68; *p* < 0.01) and IMT (*r*^2^ = 0.56; *p* < 0.05) as shown in Figure 1(C). High-VCAM-1 regions had significantly greater IMT (Figure 1(F)) and IT (Figure 1(I)) compared to Low-VCAM-1 regions supporting a relationship between local eVCAM-1 and plaque progression. Representative photomicrographs of regions of Low- and High-VCAM-1 stained for macrophage infiltration, oxidative stress (nitrotyrosine) and en-dothelial cell health (p-eNOS, eNOS) are illustrated in Figure 2. Macrophages were restricted to clusters of cells within the subendothelial layer, and were only present with High-VCAM-1 regions (Figure 2(G)). Nitrotyrosine staining within the intima was slightly more diffuse, similar to that of VCAM-1. Quantification of intimal positive staining and intensity for VCAM-1, macrophage infiltration and nitrotyrosine are shown in the 3rd and 4th rows. The percent of endothelium (data not shown) and intimal area stained for VCAM-1 and the intensity were greater in high versus Low-VCAM-1 regions (Figure 2(K) and Figure 2(P)). Moreover, regions of high- VCAM-1 had greater levels and more intense intimal SRA (Figure 2(L) and Figure 2(Q)) and nitrotyrosine (Figure 2(M) and Figure 2(R)) staining compared with Low-VCAM-1 regions (*p* < 0.05).

### eVCAM-1 is not correlated locally with markers of endothelial function

To determine if local eVCAM-1 precedes EDD, we measured expression of markers of endothelial cell function, *i.e.* p-eNOS and eNOS, in regions with Low- and High-VCAM-1 in the bifurcation. Representative images of positive staining for both markers along the carotid endothelium are illustrated in Figure 2. Endothelial p-eNOS and eNOS distribution and intensity were similar in both Low- and High-VCAM-1 regions (Figure 2(N) and Figure 2(O), Figure 2(S), Figure 2(T)). The proportion of phosphorylated to non-phosphorylated eNOS, an index of eNOS activity, was also similar for both distribution (*P* = 0.686) and intensity (*P* = 0.486) in both regions. Occasionally, diffuse eNOS staining could be seen underlying the endothelium in areas of High-VCAM-1 regions and high IMT (Figure 2(J)). We observed similar intimal eNOS staining in early atherosclerotic lesions of swine coronary arteries, but not in regions lacking intimal thickening nor in non-immune control sections (unpublished observations). Additionally, markers of endothelial function did not correlate with carotid IMT, another commonly used marker of severity of disease (IMT vs. % p-eNOS: *r*^2^ = 0.05, *p* = 0.56; IMT vs. % eNOS: *r*^2^ = 0.29, *p* = 0.16; IMT vs. p-eNOS/eNOS: *r*^2^ = 2.1 × 10^−7^, *p* = 0.99).

### eVCAM-1 is differentially distributed along the CCA and increases with age

**Figure 3:**
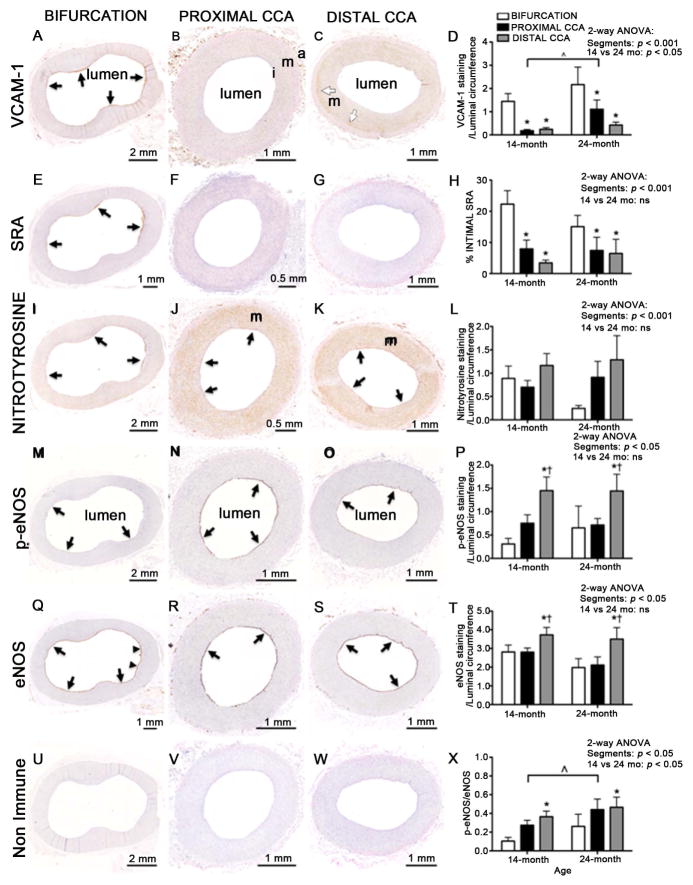
Endothelial VCAM-1, markers of atherosclerosis and endothelial health throughout carotid arteries in FH swine. Representative photomicrographs of VCAM-1 (A–C), SRA (E–G), nitrotyrosine (I–K), p-eNOS (M–O), eNOS (Q–S) and non-immune (U–W) staining in the bifurcation, proximal and distal CCA of a 14-month old animal: intima (i), media (m) and adventitia (a). In the bifurcation (A), VCAM-1 was more prominent in the intima, particularly along the endothelium (black arrows). These same regions also strongly stained for SRA (E) and nitrotyrosine (I). VCAM-1 ((B) and (C)) and SRA ((F) and (G)) in the intima (i) was markedly decreased in the proximal and distal CCA compared to the bifurcation. In contrast with VCAM-1 and SRA, nitrotyrosine was intense along the endothelium (black arrows) and in the media (m) of the proximal and distal CCA. p-eNOS (M–O) and eNOS (Q–S) were restricted to the endothelium (arrows) except in the bifurcation where faint eNOS staining was also occasionally seen in the subendothelial space (arrowheads). Quantification of endothelial VCAM-1 (D), intimal SRA (H), endothelial nitrotyrosine (L), p-eNOS (P), total eNOS (T) and the ratio of phosphorylated- to-total eNOS (p-eNOS/eNOS, X). Positive endothelial staining for all markers except SRA were normalized to the luminal circumference of the artery. SRA was expressed as a percent of intimal positive staining. eVCAM-1 (D) and intimal SRA (H) was greater in the bifurcation compared to the proximal and distal CCA. In addition, 24-month swine had greater eVCAM-1 staining compared to 14-month animals. No significant difference was seen between groups or carotid segments for endo-thelial nitrotyrosine staining. P-eNOS (P), eNOS (T) and p-eNOS/eNOS (X) were greater in the distal CCA compared to other segments. Age had no effect except on p-eNOS/eNOS which was greater at 24 months. Data are represented as mean ± SE (14 months, *n* = 14, 24 months, *n* = 10; ^*^p < 0.05 vs. bifurcation; ^†^*p* < 0.05 vs. proximal CCA; ^^^*p* < 0.05 in 24 vs. 14 months). CCA, common carotid artery; eVCAM-1, endothelial vascular cell adhesion molecule-1; SRA, Scavenger-receptor A; p-eNOS, phosphorylated eNOS; mo, month-old; ns, not statistically significant. All images were taken at 10× magnification.

Regional hemodynamics are proposed to account for heterogeneous disease development within arterial segments [11], therefore we compared the distribution of eVCAM-1 with markers of severity of disease and endothelial cell function, in the bifurcation, proximal and distal CCA of swine belonging to two different age groups, assuming that older pigs would have more severe carotid and coronary artery disease [12]. Clinical characteristics of groups are summarized in Table 1. eVCAM-1 staining was particularly intense in several areas of the bifurcation (Figure 3(A)) compared to the proximal (Figure 3(B)) and distal CCA (Figure 3(C)) where both intensity and extent of eV-CAM-1 was less. Interestingly, VCAM-1 was also moderately intense along the adventitial/medial border in the distal CCA in several pigs (Figure 3(C)). In both age groups, eVCAM-1 was greater in the bifurcation compared to the proximal and distal portion of the CCA (*p* < 0.001; Figure 3(D)) and increased at 24 months compared to 14 months (*p* < 0.05), suggesting an effect of aging on endothelial inflammation.

### eVCAM-1 is correlated with intimal macrophage accumulation, but not with oxidative stress

In the bifurcation, endothelial areas that expressed VCAM-1 also expressed greater SRA (Figure 3(E)) and nitrotyrosine (Figure 3(I)). Intimal SRA staining was markedly lower in the proximal (Figure 3(F)) and distal (Figure 3(G)) CCA compared to the bifurcation (Figure 3(E)). Thus, similar to eVCAM-1, intimal accumulation of macrophages was greater in the bifurcation compared to the proximal and distal CCA (Figure 3(H)). In contrast to VCAM-1 and SRA, nitrotyrosine staining was intense both in the endothelium as well as in the media of all carotid segments, particularly in the proximal (Figure 3(J)) and distal (Figure 3(K)) CCA and not significantly different between the three carotid segments (Figure 3(L)). Thus, eVCAM-1 was positively correlated with intimal macrophage staining in the bifurcation (*r*^2^ = 0.18, *p* < 0.05) and distal CCA (*r*^2^ = 0.33, *p* < 0.01) while no relationship between eVCAM-1 and levels of nitrotyrosine was observed.

**Figure 4:**
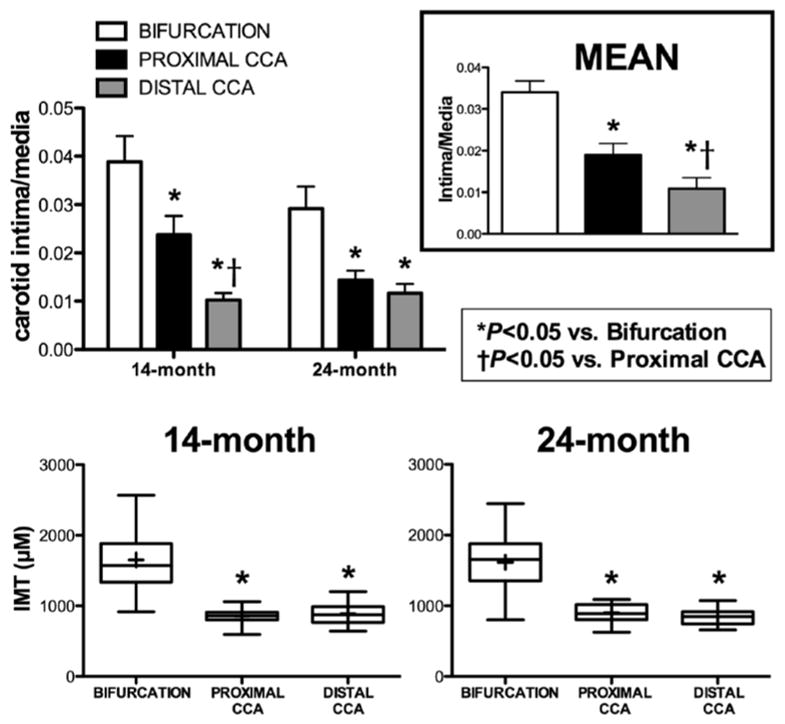
Intima-media ratio and intima-media thickness in carotid arteries of FH swine. Top left: I/M in the bifurcation, proximal and distal CCA of 14- and 24-month old FH pigs. Top right: Pooled I/M in each carotid segment from both groups. Bottom: Box plots of IMT in the three carotid segments of both age groups (box represents 25th and 75th percentile; whiskers, range; horizontal bar represents the mean. I/M and IMT were not significantly different with age. Each carotid segment I/M was different from each other (*p* < 0.001). CCA, common carotid artery; ^*^p < 0.05 vs. bifurcation; ^†^*p* < 0.05 vs. proximal CCA (14 months, n = 14; 24 months, n = 10). Values are means ± SE.

Aging did not result in changes in intimal macrophage accumulation, oxidative stress or severity of disease (IMT, I/M) despite increased VCAM-1 expression in 24 month-old pigs, suggesting that VCAM-1 may precede macrophage infiltration, oxidative stress (Figure 3(H) and Figure 3(L)) and disease (Figure 4). Regardless of age, mean I/M ratio for both groups was significantly different between carotid segments (*p* < 0.001; Figure 4 top right) while IMT was greater in the bifurcation compared to proximal and distal CCA (*p* < 0.001; Figure 4 Bottom panel).

### Total eNOS and Phosphorylated-to-total eNOS ratio is weakly correlated with eVCAM-1 in the proximal CCA

**Figure 5:**
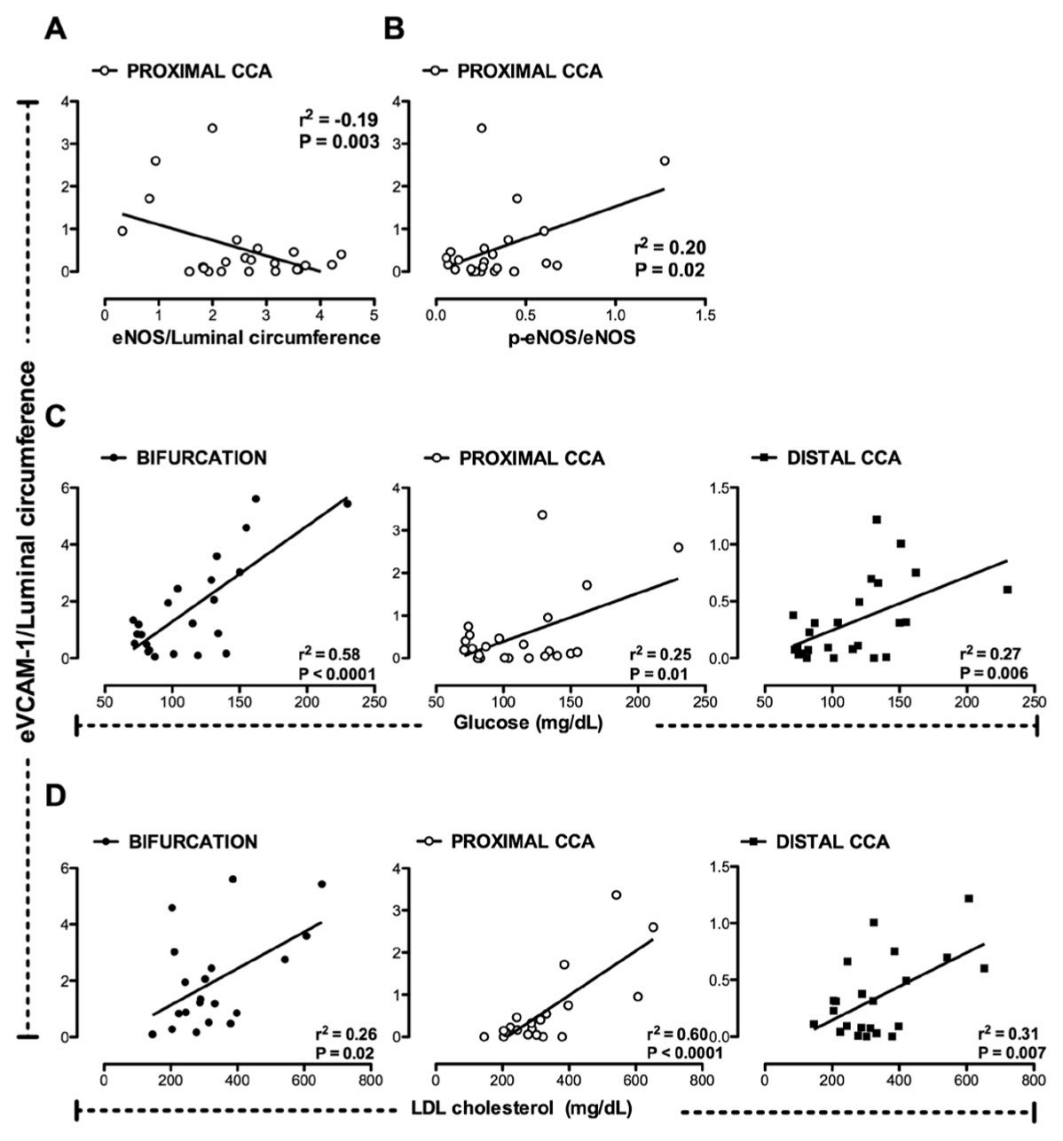
eNOS, phosphorylated-to-total eNOS ratio, glycemia, low density-lipoprotein (LDL) cholesterol and carotid eV-CAM-1 in FH swine. Endothelial VCAM-1 (eVCAM-1), and eNOS staining was expressed as the sum of endothelial positive area normalized to the luminal circumference of the artery. (A), (B): A weak negative linear correlation was found between eNOS and eVCAM-1 in the proximal CCA (A), whereas the ratio of p-eNOS/eNOS was weakly positively correlated to eVCAM-1 in the same carotid segment (B). (C), (D): A positive linear relation was found between blood glucose levels (mg/dL), LDL cholesterol (mg/dL) and endothelial VCAM-1 in all carotid segments. Using a stepwise linear regression, en-dothelial VCAM-1 in the bifurcation was best predicted by blood levels of glucose (C). LDL cholesterol best predicted eV-CAM-1 in the proximal and distal CCA (D). eVCAM-1, endothelial VCAM-1; CCA, common carotid artery; LDL, low density-lipoprotein. Significant levels were set at *α* = 0.05.

To examine the relationship between eVCAM-1 and markers of endothelial cell function, we evaluated carotid segments for p-eNOS (Figure 3(M) and Figure 3(O)) and total eNOS (Figure 3(Q) and Figure 3(S)). p-eNOS and total eNOS were significantly greater in the distal CCA compared to the bifurcation and proximal CCA (Figure 3(P) and Figure 3(T)) with no difference observed between 14- and 24-month old groups. p- eNOS/eNOS ratio, an index of eNOS activity, was increased at 24 months (*p* < 0.05; Figure 3(X)). The proportion of phosphorylated eNOS (p-eNOS/eNOS ratio) was greater in the distal CCA than the bifurcation (Figure 3(X)). Neither p-eNOS, total-eNOS nor p-eNOS/eNOS were inversely correlated to VCAM-1 in the bifurcation or distal CCA and only a weak association between eVCAM-1 and total eNOS (*r*^2^ = −0.19, *p* < 0.01, Figure 5(A)), and with p-eNOS/eNOS (*r*^2^ = −0.20, *p* < 0.05, Figure 5(B)) was present in the proximal CCA. Interes- tingly, p-eNOS, a marker of endothelial function positively correlated to EDD in humans [13] was not significantly correlated with eVCAM-1 in any of the carotid segments.

### Blood levels of glucose and total cholesterol predict eVCAM-1 in the carotid arteries

Forward stepwise regression demonstrated that blood glucose levels best predicted eVCAM-1 in the bifurcation (*r*^2^ = 0.58, *p* < 0.0001; Figure 5(C)) while LDL-C best estimated eVCAM-1 in the proximal (*r*^2^ = 0.60, *p* < 0.0001; Figure 5(D)) and distal CCA (*r*^2^ = 0.31, *p* < 0.01; Figure 5(D)). Total cholesterol was also moderately positively correlated with inflammation in all segments (data not shown). Neither HDL-C nor triglycerides level were associated with the degree of inflammation in the carotid arteries (data not shown).

### eVCAM-1 predicts CAD

**Figure 6:**
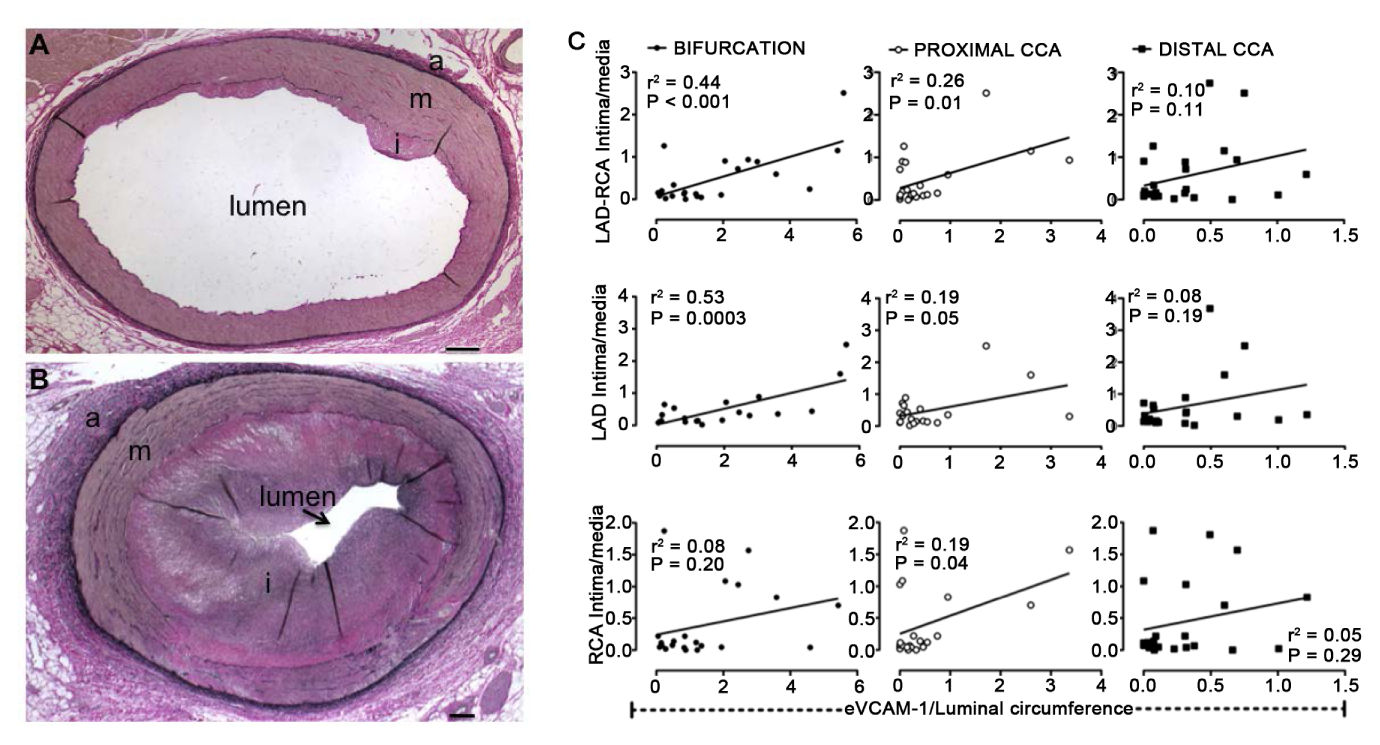
Relation between carotid eVCAM-1 and coronary artery disease (CAD). Representative images of mild (A) and severe (B) atherosclerotic lesion in the left anterior descending artery (LAD). Severity of CAD determined as mean intima- media ratio (I/M) in LAD and right coronary artery (RCA). Scale bar corresponds to 200 μM. (C) first row: A positive linear relation was found between coronary artery I/M and eVCAM-1 in the bifurcation and to a lesser extent in the proximal CCA; (C) middle row: The correlation was strongest in the LAD while eVCAM-1 was at most weakly associated with CAD in the RCA ((C), third row). eVCAM-1, endothelial VCAM-1; CCA, common carotid artery.

As anticipated, the severity of coronary atherosclerotic plaque varied greatly among our studied population, ranging from minimal to severe thickening of the arterial wall and narrowing of the lumen as illustrated in Figure 6. We compared carotid eVCAM-1 and risk factors for atherosclerosis, such as hyperglycemia, hypercholesterolemia, hypertriglyceridemia, high LDL-C, and low HDL-C, to determine which best predicted CAD. CAD was best predicted by VCAM-1 in the bifurcation (*r*^2^ = 0.44; *p* < 0.001; Figure 6 first row). When coronary arteries were examined separately, LAD disease was best predicted by eVCAM-1 in the bifurcation (*r*^2^ = 0.53; *P* < 0.001; Figure 6 middle row), while RCA disease was best correlated with LDL (*r*^2^ = 0.40; *p* < 0.01; data not shown). Additionally, no relationship was found between CAD and p-eNOS or carotid IMT, supporting the hypothesis that eVCAM-1 is a better peripheral indicator of CAD compared to endothelial function and intimal thickening.

## 4. Discussion

The purpose of this study is two-fold to determine 1) the relationship between eVCAM-1 and atherosclerosis both locally and regionally in the carotid artery and 2) the ability of carotid eVCAM-1 to predict CAD compared with other markers of atherosclerosis and of EDD. Of major importance, the current study provides the first direct evidence that carotid VCAM-1 expression is predictive of coronary atherosclerosis in a large animal model.

### Local inflammation is associated with atherosclerotic plaque progression

Our findings demonstrate that eVCAM-1 is associated with indices of atherosclerotic plaque progression, intimal macrophage accumulation, and oxidative stress locally within the carotid artery wall. In contrast, local expression of carotid eVCAM-1 is not associated with a reduction in markers of endothelial cell function, *i.e.* phosphorylated- and total-eNOS. Due to the cross-sectional design of our study, we cannot definitively conclude that local VCAM-1 expression precedes the development of lesion. However, eVCAM-1 expression varies greatly in the arterial circumference and in several regions. We observe that VCAM-1 expression is not always associated with macrophage infiltration, oxidative stress, or intimal thickening, and is consistent with VCAM-1 preceding lesion development. A longitudinal study which is similar to that done by Kaufmann *et al.* in mice [14] will be required to establish a temporal relationship between VCAM-1 and disease.

Since our study is done retrospectively and reactivity to vasodilators cannot be performed, we use phosphory-lated and total-eNOS as markers of endothelial function. Hambrecht *et al.* report a positive linear relationship between p-eNOS and acetylcholine-mediated EDD in people with stable CAD [13]. In the present study, despite the evidence of local inflammation and increased atherosclerosis in regions of High-VCAM-1, neither phospho-rylated- nor total-eNOS is reduced compared to less diseased regions. Moreover, IMT, a commonly used clinical marker for prediction of cardiovascular outcome, is not locally associated with phosphorylated-, total or phos-phorylated-to-total eNOS ratio along the arterial endothelium, indicating that inflammation precedes endothelial dysfunction and supports VCAM-1 as a better indicator of disease than assessment of endothelial function in FH carotid arteries.

Inflammation is not uniform throughout the CCA and increases with aging. The present study is the first to assess the regional distribution of an inflammatory marker along the endothelium of carotid arteries in a large animal model of spontaneous atherosclerosis. In accordance with our hypothesis, we demonstrate that eV-CAM-1 is more prevalent in the bifurcation compared to the proximal and distal CCA, which is consistent with reported increased VCAM-1 expression associated with regions of disturbed flow [15]. Regions of high shear stress, such as the central portion of the CCA, characterized by steady laminar flow [11] [16], are generally exempt of atherosclerotic lesions. In these regions, high shear stress is thought to produce nitric oxide, which in turn inhibits NF-*κ*B, an important regulator of VCAM-1 expression [17].

As seen locally in the arterial circumference, eVCAM-1 is closely associated with intimal macrophage accumulation regionally in the bifurcation and distal CCA. This is consistent with leukocyte recruitment following expression of adhesion molecules in hypercholesterolemic rabbits [18]. An association between eVCAM-1 and oxidative stress throughout the CCA is expected. However, nitrotyrosine expression is similarly elevated throughout the CCA, even in the distal segment, which has minimal expression of VCAM-1 and macrophage infiltration. These results indicate sources of free radicals and peroxynitrite other than macrophages and foam cells, for example, direct exposure of the endothelial cells to hypercholesterolemia and hyperglycemia [19] [20]. Increased oxidative stress may have overwhelmed the antioxidant effect of shear stress in the distal CCA compared to the bifurcation.

Interestingly, VCAM-1 was not inversely correlated with markers of endothelial function, aside from a weak inverse relation between eVCAM-1 and total eNOS in the proximal CCA. Regional differences were observed in the expression of phosphorylated-, total-eNOS, p-eNOS/total eNOS ratio between carotid segments. The lower phosphorylated- and total-eNOS in the bifurcation and proximal CCA compared to the distal CCA suggests a predominant role of local over systemic effects of hypercholesterolemia and hyperglycemia on endo-thelial cells. A local factor potentially responsible for the difference in levels of active and total eNOS is shear stress. Steady laminar stress activates the Akt/eNOS phosphorylation cascade, leading to nitric oxide production and EDD. Although we did not measure shear forces in our model, the central portion of the CCA where our distal segment was harvested exhibits high shear stress [21], which could explain the greater levels phosphory-lated- and total-eNOS. On the contrary, the proximity of the proximal CCA and bifurcation to the flow divider suggests turbulent flow and decreased shear stress in these segments [21] [22], which could explain the lower eNOS protein and lower activation of the Akt/eNOS phosphorylation cascade. Together, these results predict a gradient in the EDD throughout the CCA.

STEPWISE regression among several risk factors including fasting blood glucose, total-, HDL-C and LDL-C, and triglycerides, determined that blood glucose and LDL-C best predicted eVCAM-1 in the carotid arteries. The association between eVCAM-1, fasting blood glucose, and LDL-C is not surprising given that both hyper-glycemia and hypercholesterolemia are known risk factors for the development of atherosclerosis in humans and animal models [23]-[25]. Whether high fasting glucose is directly responsible for increased VCAM-1 expression in our model is unknown, although high glucose has been shown to upregulate expression of VCAM-1 in endo-thelial cells [26] [27], and soluble VCAM-1 has been correlated with disturbed glucose metabolism in men with CAD [28]. Moreover, patients with type 2 diabetes mellitus are more likely to develop CAD compared to non-diabetic patients [29]. In the settings of hypercholesterolemia, VCAM-1 is upregulated at atheroprone areas in ApoE^−/−^mice in association with plasma cholesterol [30]. Furthermore, the interaction between glucose and lipoprotein produces advanced glycosylation end products (AGEs) [31], which induce transcription factor NF-*κ*B [32] [33] and upregulation of VCAM-1 [34]. The regional differences in inflammation seen in the carotid arteries may be explained, at least in part, by the presence of local factors, such as the hemodynamics which may modulate the pro-inflammatory susceptibility of endothelial cells to systemic factors such as blood glucose and LDL-C.

Importantly we demonstrated that among several commonly used risk factors and markers of atherosclerosis, carotid eVCAM-1 best predicted the severity of CAD, particularly in the LAD, supporting a clinical use for carotid VCAM-1 in the diagnosis of CAD. Future studies will be needed to validate a similar role in humans. Interestingly, blood levels of LDL-C were superior to VCAM-1 at predicting disease in the RCA. The reason for this difference is unknown, although consistent with reports that atherosclerotic lesions in the LAD have increased inflammation than the RCA [35].

eNOS phosphorylation in the internal mammary artery has been shown to correlate with endothelial function in coronary arteries of patients with stable CAD [13]. In FH carotid arteries, no relationship was found between p-eNOS and CAD, indirectly indicating the absence of an association between peripheral EDD and CAD in our model. Therefore, we conclude that peripheral eVCAM-1 is superior to assessment of EDD at predicting CAD in FH swine. It will be imperative to determine if a similar relationship exists in human patients.

In conclusion, the current study provides the first direct evidence that carotid VCAM-1 expression is predictive of CAD in a large animal model. VCAM-1 protein expression is non-uniform both locally along the carotid arterial circumference as well as regionally throughout the common carotid artery. Heterogeneity of inflammation is likely not a unique feature to the carotid artery, stressing the importance of anatomical consistency in assessment of inflammation both longitudinally and between subjects. Importantly, carotid eVCAM-1 was the best predictor of CAD in the FH swine. Future studies are needed to determine if these findings translate to human CAD patients, but this study strongly supports the development of VCAM-1 targeted agents as non-invasive diagnostic tools to longitudinally monitor the progression of peripheral and coronary atherosclerosis.

## Figures and Tables

**Table 1:** Clinical characteristics of Familial Hypercholesterolemic (FH) swine.

Variables	Control	*n*	14 Months	*n*	24 Months	*n*
Body weight (kg)	72.0 ± 2.0	5	66.6 ± 2.9	15	110.6 ± 13.2†	10
Body fat (%)	NA	0	26.2 ± 1.5	7	27.3 ± 4.7	5
Glucose (mg/dL)	99.2 ± 3.2	0	112.1 ± 7.9	15	119.1 ± 15.1	10
Triglyceride (mg/dL)	16.4 ± 2.8	5	42.7 ± 6.3*	15	85.7 ± 7.6*†	7
Cholesterol (mg/dL)	85.6 ± 5.3	5	344.8 ± 19.6*	15	528.4 ±52.7*†	7
LDL cholesterol (mg/dL)	50.4 ± 4.6	5	270.3 ± 17.9*	15	465.0 ± 51.2*†	7
HDL cholesterol (mg/dL)	46.0 ± 1.0	5	44.2 ± 1.6	15	36.4 ± 3.0*†	7

Values for FH swine were compared to those measured in five non-FH domestic swine (control). Data are means ± SE.

* *P* < 0.05 vs. control;

† *P* < 14 vs. 24 month-old pigs. FH pigs had greater plasma triglycerides, LDL- and total cholesterol compared to control swine, whereas only 24 month-old FH pigs had lower HDL cholesterol. Body weight, cholesterol, triglyceride and LDL were increased and HDL decreased in 24- vs 14-month old Percent body fat and glucose were not significantly different between both FH groups. NA, not available; LDL indicates low-density lipoprotein cholesterol; HDL, high-density lipoprotein cholesterol.
